# Basal Synaptic Transmission and Long-Term Plasticity at CA3-CA1 Synapses Are Unaffected in Young Adult PINK1-Deficient Rats

**DOI:** 10.3389/fnins.2021.655901

**Published:** 2021-08-13

**Authors:** Adeel A. Memon, Micah E. Bagley, Rose B. Creed, Amy W. Amara, Matthew S. Goldberg, Lori L. McMahon

**Affiliations:** ^1^Department of Neurology, The University of Alabama at Birmingham, Birmingham, AL, United States; ^2^Department of Neuroengineering, School of Engineering, The University of Alabama at Birmingham, Birmingham, AL, United States; ^3^Center for Neurodegeneration and Experimental Therapeutics, The University of Alabama at Birmingham, Birmingham, AL, United States; ^4^Department of Cell, Developmental, and Integrative Biology, The University of Alabama at Birmingham, Birmingham, AL, United States; ^5^Department of Neurobiology, The University of Alabama at Birmingham, Birmingham, AL, United States

**Keywords:** Parkinson’s disease, PINK1, hippocampus, CA3-CA1 synapses, long term plasticity, basal synaptic transmission

## Abstract

Loss of function mutations in PARK6, the gene that encodes the protein PTEN-induced kinase 1 (PINK1), cause autosomal recessive familial Parkinson’s disease (PD). While PD is clinically diagnosed by its motor symptoms, recent studies point to the impact of non-motor symptoms, including cognitive dysfunction in the early pre-motor stages of the disease (Aarsland et al., 2004; Chaudhuri and Schapira, 2009). As the hippocampus is a key structure for learning and memory, this study aimed to determine whether synaptic transmission is affected at CA3-CA1 excitatory synapses in PINK1 knockout rats at an age when we recently reported a gain of function at excitatory synapses onto spiny projection neurons in the dorsal striatum (Creed et al., 2020) and when motor symptoms are beginning to appear (Dave et al., 2014). Using extracellular dendritic field excitatory postsynaptic potential recordings at CA3-CA1 synapses in dorsal hippocampus 4-to 5- month old PINK1 KO rats and wild-type littermate controls, we observed no detectable differences in the strength of basal synaptic transmission, paired-pulse facilitation, or long-term potentiation. Our results suggest that loss of PINK1 protein does not cause a general dysfunction of excitatory transmission throughout the brain at this young adult age when excitatory transmission is abnormal in the striatum.

## Introduction

Human Parkinson’s disease (PD) affects a variety of brain regions, leading to multiple motor and non-motor symptoms. Cognitive impairment is a disabling non-motor symptom, and affects approximately 25% of newly diagnosed PD patients (Ibarretxe-Bilbao et al., 2012). As the disease advances, up to 80% of PD patients without prior cognitive dysfunction develop mild cognitive impairment (PD-MCI) and dementia (Hely et al., 2008). Consequentially cognitive deficits have important implications in the disease management. Unfortunately, there are no effective therapeutic options available due to the incomplete understanding of underlying synaptic mechanisms leading to cognitive dysfunction.

In recent years, the field of movement disorders has evolved from the conventional idea that hippocampal dysfunction plays a minor role in PD, to one that now regards deficits in hippocampal synaptic plasticity to contribute significantly to memory loss in PD. This concept is supported by studies showing hippocampal atrophy in PD patients with impaired cognition (Camicioli et al., 2003; Bruck et al., 2004; Calabresi et al., 2013; Kandiah et al., 2014), and studies showing lower baseline volume in the CA1 hippocampal region accompanied by deficits in baseline attention in PD patients with MCI. Longitudinally, the decline in episodic memory appears to be associated with increasing atrophy of CA2-CA3 regions over 18 months (Foo et al., 2017). Thickness of hippocampal CA1 stratum pyramidale is also associated with episodic memory impairment in PD patients (Boentert, 2019). Importantly, in neurodegenerative diseases such as PD, synaptic transmission is negatively impacted prior to overt structural and behavioral abnormalities (Wishart et al., 2006; Raymond et al., 2011). Thus, exploration of changes in synaptic networks using preclinical models with a well-established timeline of motor deficits can provide insight into how relevant synaptic circuits are altered prior to and after motor symptoms appear.

In this study we leveraged the recently developed PD rat model with loss of function mutation in *PARK6*, the gene that encodes the protein PTEN-induced kinase 1 (PINK1) (Valente et al., 2004), to study hippocampal excitatory transmission. In humans, loss of function PINK1 mutations cause an autosomal recessive early-onset form of PD with clinical symptoms similar to idiopathic PD (Kasten et al., 2010). However, the mechanism by which PINK1 deficiency causes PD is unknown. Under physiological conditions, PINK1 is involved in many functions such as mitochondrial autophagy (Kane et al., 2014; Lazarou et al., 2015; Truban et al., 2017) and bioenergetics (Rango et al., 2020), maintenance of mitochondrial calcium homeostasis (Heeman et al., 2011), and misfolded protein clearance (Du et al., 2017), plus neuronal branching (Dagda et al., 2014), regulation of adaptive immunity (Matheoud et al., 2019) and neuroinflammation (Sliter et al., 2018). In a mouse model of Alzheimer’s disease, PINK1 overexpression rescued impairments in hippocampal LTP (Du et al., 2017). However, in PINK1 KO mice, no changes were detected in hippocampal LTP (Feligioni et al., 2016). The occurrence of both motor and non-motor phenotypes in PINK1 KO rats, specifically at ages prior to reported nigral cell loss, prompted our investigation of possible impairments in hippocampal synaptic transmission. Using PINK1 KO rats, we explored how loss of PINK1 impacts synaptic function at hippocampal CA3-CA1 synapses as this region represents the primary experimental model for the synaptic changes underlying learning and memory. We chose to perform these experiments in rats at 4 to 5 months of age because our laboratory previously found striatal circuit dysfunction at this age when motor symptoms begin to appear but prior to the age when dopaminergic neuronal loss becomes apparent (Creed et al., 2020).

## Materials and Methods

### Animals

PINK1 KO rats on a Long-Evans background were obtained from Horizon Discovery with a breeding license and bred in-house at the University of Alabama at Birmingham (UAB) to obtain homozygous PINK1 KO and wild-type (WT) littermate controls (Dave et al., 2014). All breeding and experimental procedures were performed per the National Institutes of Health Guide for the Care and Use of Laboratory Animals with prior review and approval by the UAB Institutional Animal Care and Use Committee. Rats were provided food and water *ad libitum*, maintained on a 12-h light/dark cycle with lights on at 6 AM, 22°C, 50% humidity, and all standard laboratory conditions. Male rats were housed in same-sex groups and aged to 4 to 5 months for all experiments.

### Slice Preparation

Between 7 and 9 am, rats were anesthetized using isoflurane, rapidly decapitated and brains removed. 400 μm thick coronal slices encompassing the dorsal hippocampus were prepared using a vibratome (Leica VT 1000P). For input-output (I/O) curves and paired-pulse ratio (PPR) experiments, slices were sectioned in ice-cold, high sucrose, artificial cerebrospinal fluid (aCSF) containing (in mM: 85.0 NaCl, 2.5 KCl, 4.0 MgSO_4_ × 7 H_2_0, 0.5 CaCl_2_ × 2 H_2_0, 1.25 NaH_2_PO_4_, 25.0 NaHCO_3_, 25.0 glucose, 75.0 sucrose) to preserve neuronal health and limit excitotoxicity. For long-term potentiation experiments, slices were prepared in aCSF containing, in mM: [119.0 NaCl, 2.5 KCl, 1.3 MgSO_4_, 2.5 CaCl_2_, 1.0 NaH_2_PO_4_, 26.0 NaHCO_3_, 11.0 Glucose (saturated with 95% O2, 5% CO2, pH 7.4)]. Before transferring to the recording chamber for electrophysiology experiments, slices were recovered in a submersion chamber for at least 60 min in regular aCSF.

### Electrophysiology

Extracellular field excitatory postsynaptic potentials (fEPSPs) were measured from the dendritic region of CA1 pyramidal cells following stimulation of CA3 Schaffer collateral axons in dorsal hippocampus. All data were acquired with an Axopatch 200B amplifier, Digidata 1440A, and data acquisition software pClamp 10 (Molecular Devices, San Jose, CA, United States). Correct electrode placement for baseline fEPSPs was confirmed by the generation of paired-pulse facilitation (PPF) characteristic of CA3-CA1 synapses (Wu and Saggau, 1994). Schaffer collaterals were stimulated using insulated twisted nichrome wire (A-M Systems, Inc., Seqium, WA, United States) or tungsten electrodes (FHC, Frederick Haer and Co, ME, United States) placed in CA1 stratum radiatum within 200–300 μm of an aCSF filled glass recording electrode. Baseline fEPSPs (∼50% of maximal response) were recorded by delivering 0.1 Hz stimulation for 100 μs to generate fEPSPs of ∼0.5 mV in amplitude.

Input/Output Curves (I/O) were obtained after a stable 10-min baseline recording. The curves were produced by gradually increasing the stimulus intensity in 10 μA increments until it reached 200 μA intensity, which generated the maximal fEPSP slope. Ten fEPSP events collected at a single stimulus strength were averaged and plotted as a single fEPSP slope for each stimulus intensity. Data collected from multiple slices from an individual animal were averaged together to generate I/O curves representing the data from that single animal.

Paired-Pulse Ratio (PPR) was generated using pairs of stimuli delivered at inter-stimulus interval (ISIs) of 10, 20, 50, 150, 200, and 400 milliseconds (ms). The ratio was calculated by dividing the slope of the second event by the slope of the first event. Like I/O, data collected from multiple slices from an individual animal were averaged to represent data from that animal.

Long-term potentiation was induced following a 20-min stable baseline, using either eight bouts of theta-burst stimulation (strong TBS) or four bouts of theta-burst stimulation (weak TBS) with each round consisting of 5 pulses at 100 Hz repeated 10× at 200 ms intervals, and each bout separated by 20 ms (Barnes et al., 1996; Watabe and O’Dell, 2003; Kumar et al., 2007).

### Statistical Analysis

GraphPad Prism 8 software was used for all statistical analyses and graphing. The researcher performing the slice electrophysiology experiments was blind to genotype, which was disclosed only at the final analysis. All data were analyzed using Repeated measures two-way ANOVA or paired *t*-test as appropriate. All results are reported as mean + SEM with significance set at a *p*-value of less than 0.05 (^∗^).

## Results

### Basal Synaptic Transmission Is Not Altered at CA3-CA1 Synapses in PINK1 KO Rats

**FIGURE 1:**
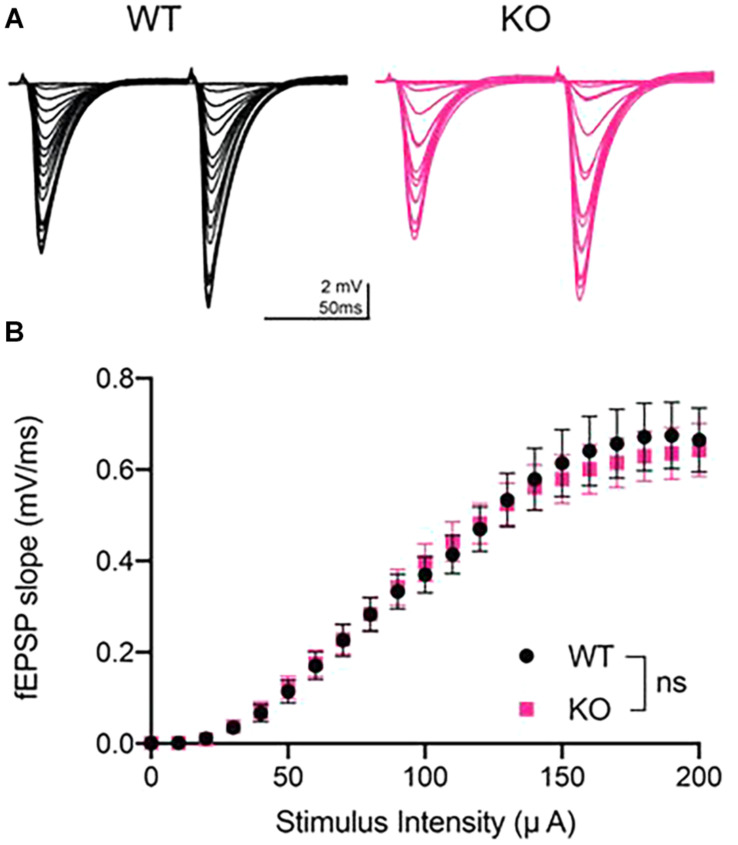
Input/output curves showed no difference in basal synaptic strength at CA3-CA1 in PINK1 KO rats compared to WT littermate controls. **(A)** Representative fEPSP traces from 4 months WT (black) and PINK1 KO (Pink) rats. **(B)** After a stable 10 min baseline, input-output (I/O) curves were obtained by increasing the stimulus intensity (10 μA increments) until a maximal fEPSP slope was obtained, usually at 200 μA stimulus intensity. Initial slope of the ten fEPSPs generated at each stimulus intensity were averaged and plotted as a single value. No statistical difference (*p* > 0.05) was found after using repeated measures two-way ANOVA [*F*_(1,50)_ = 0.01699] at the maximal stimulus intensity between WT (*n* = 26 slices/11 animals) and PINK1 KO rats (*n* = 26 slices/11 animals).

We generated fEPSP I/O curves at CA3-CA1 synapses to determine whether maximal synaptic strength is decreased in PINK1 KO rats compared to WT littermate controls at 4 to 5 months of age. I/O curves were obtained by incrementally increasing stimulus intensity from 0 to 200 μA in 10 μA intervals (Figure 1A). We found no significant difference in basal synaptic transmission between the two genotypes (*p* > 0.05; Repeated measures two- way ANOVA, Figure 1B). This finding shows that synaptic connectivity in area CA1 is not altered by loss of PINK1 protein at 4 to 5 months of age, the same age at which we have reported heightened excitatory transmission in dorsal striatum (Creed et al., 2020).

### Paired-Pulse Ratio Is Normal in PINK1 KO Rats

**FIGURE 2:**
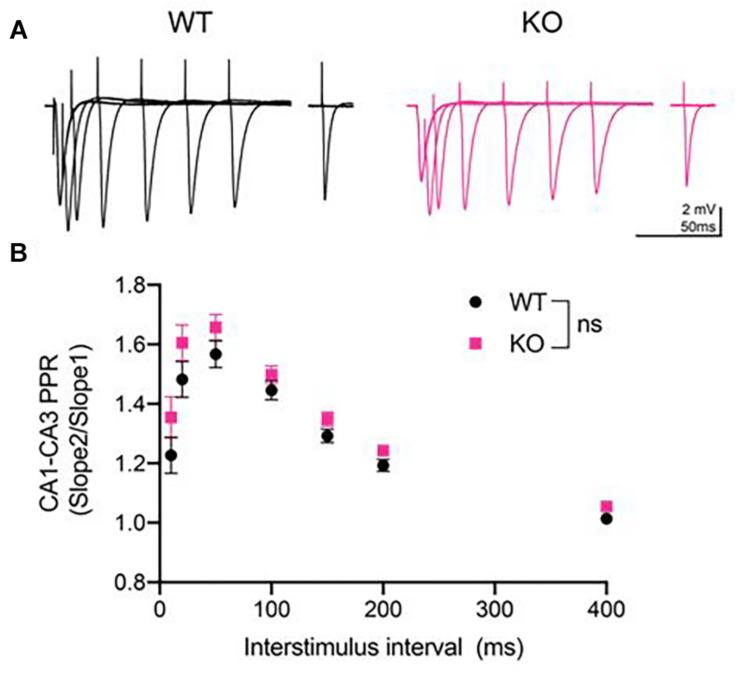
Paired-pulse ratio (PPR) was unaltered at CA3-CA1 synapses in PINK1 KO rats compared to WT littermate controls. **(A)** Representative fEPSP traces from 4 months WT (black) and PINK1 KO (Pink) rats. **(B)** After a 10 min stable baseline, pairs of stimulation were delivered at 10, 20, 50, 150, 200, and 400 milliseconds (ms) inter-stimulus intervals (ISIs). PPR was calculated by dividing the initial slope of the first event. Repeated measures two-way ANOVA showed no statistical differences [*p* > 0.05, *F*_(1,54)_ = 2.418] between WT (*n* = 26 slices/11 animals) and PINK1 KO rats (*n* = 26 slices/11 animals).

Next, we measured the PPR, which is an indirect measure of presynaptic neurotransmitter release probability (Dobrunz and Stevens, 1997). Paired-pulse stimulation generates PPF at CA3-CA1 synapses, as these synapses have low initial release probability (Dobrunz and Stevens, 1997). We analyzed PPR at 10, 20, 50, 100, 150, 200, and 400 ms inter-stimulus interval (Figure 2A) and found no significant difference between genotypes at any inter-stimulus interval (*p* > 0.05, Repeated measures two- way ANOVA, Figure 2B).

### Long-Term Potentiation Is Not Different Between WT and PINK1 KO Rats

**FIGURE 3:**
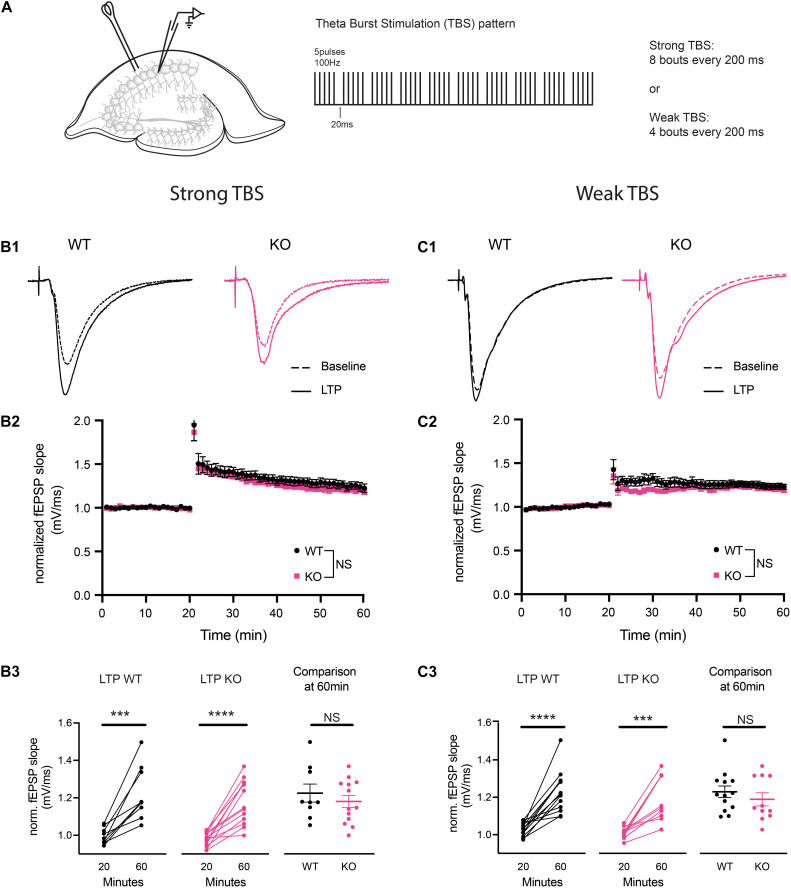
Theta burst stimulation (TBS) induced long-term potentiation (LTP) was not different at CA3-CA1 synapses in PINK1 KO compared with WT littermate controls. **(A)** Schematic of stimulating and recording electrode in area CA1 of the hippocampus and the TBS pattern used to produce LTP (5 pulses of 100 Hz, each bout separated by 20 ms, repeated 10 times with 200 ms interstimulus interval). Initially, we performed LTP induction using a strong (8 bouts) TBS. To evaluate whether a strong TBS produced a ceiling effect at these synapses, we then induced LTP using a weak (4 bouts) TBS. Panels **(B1,C1)** show representative fEPSP traces from baseline and LTP induced by both strong and weak TBS from 4 months WT (black) and PINK1 KO (Pink) rats. Panels **(B2,C2)** show NMDA receptor (NMDAR) dependent LTP induction at CA3-CA1 synapses, following a 20 min stable baseline, using a strong TBS or a weak TBS. No statistical difference (*p* > 0.05) was found after using Repeated measures two-way ANOVA [strong TBS: *F*(1,18) = 0.7216; weak TBS: *F*(1,22) = 1.301] between WT littermate controls (strong TBS: *n* = 9 slices/6 animals; weak TBS: *n* = 13 slice/4 animals) and PINK1 KO rats (strong TBS: *n* = 11 slices/7 animals; weak TBS: *n* = 11 slices/3 animals). Panels **(B3,C3)** show comparison of fEPSP slope at 20 min baseline and 60 min post TBS successfully induced LTP in both WT (strong TBS: *p* = 0.0007, paired *t*-test, *t* = 5.301, *df* = 8; weak TBS: *p* = <0.0001, paired *t*-test, *t* = 6.366, *df* = 12) and PINK1 KO (strong TBS: *p* = <0.0001, paired *t*-test, *t* = 6.542, *df* = 12; weak TBS: *p* = 0.0002 paired *t*-test, *t* = 5.549, *df* = 10) rats. However, no change was detected between WT and PINK1 KO rats when compared at 60 min post LTP induction (strong TBS: *p* = 0.4299; Unpaired Student’s *t*-test, *t* = 0.8056, *df* = 20; weak TBS: *p* = 0.3980; unpaired Student’s *t*-test, *t* = 0.8619, *df* = 22).

To determine the ability of CA3-CA1 synapses to undergo long-term plasticity at 4 to 5 months of age in PINK1 KO rats, we investigated LTP at CA3-CA1 synapses. Initially, we used a strong TBS stimulation to induce LTP. Comparison of averaged baseline fEPSP slope measured from last 6 sweeps of a stable 20 min recording to the averaged fEPSP slope from the last 6 sweeps at 60 min post-tetanus showed significant LTP in WT (*p* = 0.0007, paired *t*-test, *t* = 5.301, *df* = 8) and PINK1 KO (*p* ≤ 0.0001, paired *t*-test, *t* = 6.542, *df* = 12) (Figure 3 panel B3), with no significant differences in LTP magnitude between groups [*p* > 0.05, Repeated measures two-way ANOVA, *F* (1,18) = 0.7216, Figure 3 panel B2]. To rule out the possibility that strong TBS might have masked a difference in LTP magnitude between the two groups, we next asked whether a difference could be observed using a weaker TBS stimulation. Comparison of baseline fEPSP slope measured at 20 min to the averaged fEPSP slope measured at 60 min post-weak TBS showed significant LTP in WT (*p* ≤ 0.0001, paired *t*-test, *t* = 6.366, *df* = 12) and PINK1 KO (*p* = 0.0002, paired *t*-test, *t* = 5.549, *df* = 10) (Figure 3 panel C3), with no significant differences in LTP magnitude between groups (*p* > 0.05; Repeated measures two- way ANOVA, *F* (1,22) = 1.301, Figure 3 panel C2). This suggests that there was no ceiling effect from strong TBS, confirming that long-term plasticity is not altered in PINK1 KO rats compared to WT littermates.

## Discussion

The goal of this study was to determine whether hippocampal excitatory transmission in PINK1 KO rats is altered compared to WT rats at an age when motor and non-motor phenotypes are just appearing prior to reported nigral cell loss and when we have observed an increase in excitatory transmission in the dorsal striatum (Creed et al., 2020). We found no detectable changes in the strength of basal synaptic transmission, short-term presynaptic plasticity or LTP at CA3-CA1 synapses in PINK1 rats compared to WT rats. These results indicate that synaptic function is not significantly impacted by loss of PINK1 protein at this young adult age.

First, we investigated the strength of synaptic transmission at CA3-CA1 synapses by measuring maximum transmission using I/O curves and found no genotype differences. Similar results were observed in PINK1 KO mice where no change was observed in I/O curves at CA3-CA1 synapses in two and six-month-old animals. However, the frequency, but not amplitude, of spontaneous excitatory postsynaptic currents (sEPSCs) was increased at CA3-CA1 synapses at 6 months of age in PINK1 KO mice (Feligioni et al., 2016). This finding is similar to our finding of increased frequency and amplitude of spontaneous EPSCs recorded from striatal spiny projection neurons in PINK1 KO rats at 4 months of age, in the absence of a change in the corticostriatal I/O curve (Creed et al., 2020). In contrast to PINK1 KO rats, no difference was observed in PINK1 KO mice in the frequency or amplitude of spontaneous EPSCs or miniature EPSCs recorded from spiny projection neurons in dorsal striatum (Madeo et al., 2014). Further investigations of CA1 pyramidal neurons in PINK1 KO rats using whole-cell patch clamp is needed in future experiments to investigate whether there is a similar increase in frequency of spontaneous EPSCs, or other alterations in synaptic transmission not observed in the current study.

To determine if there was an alteration in presynaptic release probability, we measured the PPR and did not find any statistical difference between genotypes at any inter-stimulus interval. A similar result was reported in an analysis of PINK1 KO mice (Feligioni et al., 2016). Specifically, no changes in PPR between PINK1 KO and WT mice at 2 and 6 months of age was observed, although an increase in the frequency of the spontaneous EPSCs was found. The authors speculated that this effect on spontaneous EPSCs might be linked to increased presynaptic accumulation of alpha-synuclein. Previous studies from our lab and others reported the spontaneous appearance of proteinase K-resistant α-synuclein-immunoreactive aggregates in various brain regions of PINK1 KO rats, including cortex, thalamus, striatum, and ventral midbrain (Grant et al., 2015; Creed and Goldberg, 2018). Recently, using slice electrophysiology, we reported increased glutamate transmission onto dorsal striatum spiny projection neurons in PINK1 KO rats at 4 months of age (Creed et al., 2020). α-synuclein plays a vital role in the presynaptic mobilization of the reserve pool of neurotransmitter vesicles, not only for dopamine but also for glutamate (Gureviciene et al., 2007). Our inability to detect changes in short-term synaptic plasticity in acute hippocampal slices of PINK1 KO rats may be related to the apparent lack of α-synuclein pathology in this area at this age.

We found no impact of the loss of PINK1 on LTP induced either by strong or weak TBS at CA3-CA1 synapses. This suggests that these synapses do not differ in their ability to undergo long-term plasticity between the two genotypes, and there was no saturation effect from strong TBS. In transgenic mice expressing a 120 amino acid truncated form of α-synuclein, there is a reduction in striatal dopamine levels and impaired ability to generate hippocampal CA1 LTP (Tofaris et al., 2006; Costa et al., 2012). In both homozygous and heterozygous PINK1 KO mice, evoked dopamine release was decreased, leading to impaired corticostriatal LTP (Kitada et al., 2007; Madeo et al., 2014). Consistent with this, we have previously reported decreased dopamine tone in PINK1 KO rats at corticostriatal synapses at 4 months of age and decreased striatal dopamine levels at age 12 months compared to 4 months, measured using *in vivo* microdialysis (Creed et al., 2019; Creed et al., 2020). Because bidirectional plasticity is critical for normal hippocampus dependent learning and memory (Wang et al., 2003), it will be important to determine in future studies whether LTD is intact at hippocampal excitatory synapses in PINK1 KO rats, and how loss of PINK1 during the aging process alters long-term plasticity that contributes to learning and memory.

In conclusion, this short report is the first hippocampal synaptic physiology study to investigate the impact of PINK1 deficiency in acute brain slices from PINK1 KO rats. By recording extracellular dendritic fEPSPs, we showed no early functional changes in mechanisms of short- and long-term plasticity as well as the strength of basal synaptic transmission at CA3-CA1 hippocampal synapses. These results demonstrate that loss of PINK1 does not alter hippocampal synaptic plasticity at onset of both motor and non-motor phenotypes.

## Data Availability Statement

The original contributions presented in the study are included in the article/supplementary material, further inquiries can be directed to the corresponding author/s.

## Ethics Statement

The animal study was reviewed and approved by the UAB Institutional Animal Care and Use Committee.

## Author Contributions

AM conceptualized the study, performed the majority of the experiments and analyses, made the figures, and wrote the first draft of the manuscript. MB and RC performed some experiments, updated the figures, performed the statistics, and edited the manuscript. LM, AA, and MG conceptualized the study, reviewed, and edited the manuscript. All authors approved the submitted version.

## Conflict of Interest

The authors declare that the research was conducted in the absence of any commercial or financial relationships that could be construed as a potential conflict of interest.

## Publisher’s Note

All claims expressed in this article are solely those of the authors and do not necessarily represent those of their affiliated organizations, or those of the publisher, the editors and the reviewers. Any product that may be evaluated in this article, or claim that may be made by its manufacturer, is not guaranteed or endorsed by the publisher.
